# Ultra-processed foods: how functional is the NOVA system?

**DOI:** 10.1038/s41430-022-01099-1

**Published:** 2022-03-21

**Authors:** Véronique Braesco, Isabelle Souchon, Patrick Sauvant, Typhaine Haurogné, Matthieu Maillot, Catherine Féart, Nicole Darmon

**Affiliations:** 1 VAB-Nutrition, 63100 Clermont-Ferrand, France; 2 Avignon University, INRAE, UMR SQPOV, F-84000 Avignon, France; 3 University Bordeaux, UMR CNRS 5248, CBMN, 33600 Pessac, France; 4 Feed & Food Department, Bordeaux Sciences Agro, 33175 Gradignan, France; 5 MS-Nutrition, 13385 Marseille, France; 6 University Bordeaux, INSERM, BPH, U1219, F-33000 Bordeaux, France; 7 MoISA, University Montpellier, CIRAD, CIHEAM-IAMM, INRAE, Institut Agro, IRD, Montpellier, France

**Keywords:** Scientific community, Epidemiology

## Abstract

**Background:**

In the NOVA classification system, descriptive criteria are used to assign foods to one of four groups based on processing-related criteria. Although NOVA is widely used, its robustness and functionality remain largely unexplored. We determined whether this system leads to consistent food assignments by users.

**Methods:**

French food and nutrition specialists completed an online survey in which they assigned foods to NOVA groups. The survey comprised two lists: one with 120 marketed food products with ingredient information and one with 111 generic food items without ingredient information. We quantified assignment consistency among evaluators using Fleiss’ κ (range: 0–1, where 1 = 100% agreement). Hierarchical clustering on principal components identified clusters of foods with similar distributions of NOVA assignments.

**Results:**

Fleiss’ κ was 0.32 and 0.34 for the marketed foods (*n* = 159 evaluators) and generic foods (*n* = 177 evaluators), respectively. There were three clusters within the marketed foods: one contained 90 foods largely assigned to NOVA4 (91% of assignments), while the two others displayed greater assignment heterogeneity. There were four clusters within the generic foods: three clusters contained foods mostly assigned to a single NOVA group (69–79% of assignments), and the fourth cluster comprised 28 foods whose assignments were more evenly distributed across the four NOVA groups.

**Conclusions:**

Although assignments were more consistent for some foods than others, overall consistency among evaluators was low, even when ingredient information was available. These results suggest current NOVA criteria do not allow for robust and functional food assignments.

## Introduction

There is increasing evidence that foods are not the simple sum of their nutrients [1]. When characterizing foods, it is essential to consider factors such as processing and formulation, which have grown more and more complex over the years. Whether carried out within households, artisanal settings, or factories, food processing aims to ensure product safety, digestibility, and palatability. It also seeks to improve shelf life and simplify meal preparation [2]. Human diets are progressively incorporating larger quantities of industrially processed foods [3]. At present, several systems are used to classify foods according to processing-related criteria [4–10] each employing different criteria and metrics.

NOVA is, by far, the most common of such systems [9]. Its stated purpose is to classify “all foods according to the nature, extent, and purposes of the industrial processes they undergo” [10]. In the NOVA system, foods are assigned to one of four groups: (i) NOVA1 contains “unprocessed or minimally processed foods,” namely the edible parts of plants or animals that have been taken straight from nature or that have been minimally modified/preserved; (ii) NOVA2 contains “culinary ingredients,” such as salt, oil, sugar, or starch, which are produced from NOVA1 foods; (iii) NOVA3 contains “processed foods,” such as freshly baked breads, canned vegetables, or cured meats, which are obtained by combining NOVA1 and NOVA2 foods; and (iv) NOVA4 contains “ultra-processed foods,” namely ready-to-eat industrially formulated products that are “made mostly or entirely from substances derived from foods and additives, with little if any intact Group 1 food” [9].

Nutritional epidemiologists are increasingly using NOVA to explore relationships among the consumption of highly processed foods and diet quality or health outcomes. Indeed, NOVA was used in 95% of the studies on this topic published between 2015 and 2019, and which have been included in a recent systematic review [11]. Furthermore, policymakers are moving to use NOVA assignments to guide public health decisions. For example, several Latin America countries have constructed dietary guidelines based on using NOVA [12, 13], and the French government is drawing upon NOVA in its objective to reduce ultra-processed food consumption by 20% [14].

It thus seems likely that NOVA will be employed in an ever-broader range of contexts. Nevertheless, aside from some sparse past work [15, 16], the system’s robustness, functionality, and consistency remain poorly characterized. Because its classification approach is purely descriptive in nature, it opens the door to ambiguity and differences in interpretation [17]. Indeed, even experts face difficulties and have disagreements when employing it [18–20].

Here, we explored the robustness and functionality of the NOVA classification system by determining whether a large number of food and nutrition specialists arrived at consistent food assignments when applying the system’s criteria. We also differences in assignments among evaluators and the relationships between NOVA assignments and food nutritional quality based on known nutrient profiling systems.

## Methods

A schematic of our experimental design is presented in Supplementary Fig. 1. We invited food and nutrition specialists to take part in a survey via an online interface that we developed. The survey set-up was as follows: the individuals taking the survey (hereafter, evaluators) were given a description of the NOVA classification system and its food assignment criteria. Then, the evaluators indicated whether they wanted to assess one or two lists of foods. In this study, we defined an “assignment” as the act of assigning a food to one of the four NOVA groups (NOVA1, NOVA2, NOVA3, or NOVA4). Evaluators were also asked to rate their level of confidence in each of their assignments. Using these data, we explored the relationships between the most common assignment (NOVA_maj_) made by the evaluators (e.g., NOVA1_maj_ = the most common assignment for a given food was NOVA1) and food nutritional quality. The latter was determined using several nutrient profiling systems.

### Food lists

One list containing 120 foods (hereafter, marketed foods) that were accompanied by detailed ingredient information. These marketed foods came from an official database of commercially available packaged foods in France [21]. We focused on three categories of foods, namely fresh dairy products, bread products, and mixed dishes, because they contain marketed foods commonly consumed in France [22] and are thought to display diversity in recipes and formulations. Forty food products were randomly selected from each category, using a weighted approach to ensure product representativeness within categories (e.g., the number of sandwich breads in the sample reflected the proportion of sandwich breads within the bread product market as a whole). The products were identified using generic descriptors; no brand names were employed for reasons of confidentiality. From the database, we also acquired information on the foods’ ingredients (including food additives) and nutrient content (i.e., as presented on food packaging).

One list containing 111 foods (hereafter, generic foods) that came from a dietary survey that was performed as part of the Three-City Study (Bordeaux cohort) and that combined a food frequency questionnaire with a 24-h dietary recall approach [23, 24]. We identified the most frequently consumed food based on the 24-h recall findings for each of the 54 FFQ food categories (e.g., apples were the most frequently consumed fruit); this information was used to create a list of 54 generic foods.

So that both lists were of similar size and structure, the list of generic foods was expanded by adding foods from the dairy (*n* = 22), bread (*n* = 13), and mixed dish (*n* = 22) categories using the 24-hour recall data. In other words, while the list of marketed foods included a wide range of products from three categories, the list of generic foods contained a few foods from multiple food categories. Additional information about the foods in both lists, and foods which overlapped between the two lists, are available in the supplementary materials (see Supplementary Tables 1 and 2).

### Evaluators

Since NOVA is mostly used by specialists, we specifically attempted to invite evaluators with at least a basic background in nutrition and/or the food sciences. We targeted four main groups: researchers with expertise in human nutrition, researchers with expertise in food technology, health professionals who provide nutritional guidance (i.e., medical doctors and dieticians), and skilled research and development professionals working in the food industry. We invited people to take part in the study by directly contacting scientific and/or clinical societies, research institutes, and professional associations; we requested that our invitation be restricted to their professional networks. Anyone wishing to participate could immediately log into the online survey interface; the names and affiliations of evaluators were kept fully anonymous, as per the European General Data Protection Regulation and French regulatory requirements. The exact number of invited professionals was not available.

### Online survey

The survey could be accessed from November 27, 2019 to February 8, 2020. The first page presented the survey’s objective. It was followed by a thorough description of the NOVA classification system and its food assignment criteria, directly translated into French from the two original articles written by NOVA’s creators [9, 10]. Links were provided to these publications and to a list of all the additives used in Europe (E number and technological functions). The entire survey is available in the online supplementary materials (OSM1 to OSM4).

Evaluators were first asked to self-assess their expertise in human nutrition and food technology using a Likert scale (0–6) and to indicate whether they wanted to work on one or both food lists. If evaluators wanted to work on a single list, they were given List of marketed foods or List of generic foods at random. If evaluators wanted to work on both lists, List of marketed foods and List of generic foods appeared in a randomized order. In each list, foods were presented in blocks (i.e., five per page). Food occurrence within a given block was random, and blocks were presented at random. Returning to previous pages was not possible. Evaluators were asked to assign each food to a NOVA group and then rate their level of confidence in their assignment, on a scale from low to high (four levels). We ran a pilot version of the survey using 10 outside volunteers who represented the different types of desired evaluators. The goal was to verify survey feasibility and to estimate the time needed for its completion (~1 h/list).

### Nutrient profiling

At present, several systems are used to assess food nutritional quality. Here, we employed the Nutri-Score system [25], the SAIN,LIM system [26], and the Nutrient Rich Food (NRF) Index (version 9.3) [27, 28] to generate profiles for each food in the two lists; we also estimated their energy density levels (kcal/100 g). We obtained the nutritional information for these calculations from the OQALI database [21] and the CIQUAL database;[29] when a food was absent from the databases, we used the information for the most similar food that was present.

Briefly, the Nutri-Score system considers a food’s levels (per 100 g) of more beneficial nutrients (i.e., protein, fiber, and percentages of fruits, nuts, vegetables, olive oil, canola oil, and walnut oil) and less beneficial nutrients (i.e., energy, total sugar, sodium, and saturated fat). The food is then assigned to one of five classes, which range from A (highest nutritional quality) to E (lowest nutritional quality). In the SAIN,LIM system, SAIN stands for “score of nutritional adequacy of individual foods” and expresses the density of five beneficial nutrients (i.e., protein, fiber, vitamin C, calcium, and iron) per 100 kcal of a food. LIM stands for “limit” and expresses the levels of three less beneficial nutrients (sodium, free sugars, and saturated fatty acids) per 100 g of a food. Using thresholds for each score, four classes can be defined: 1 = high SAIN, low LIM (the best class); 2 = low SAIN, low LIM; 3 = high SAIN, high LIM; and 4 = low SAIN, high LIM (the worst class) [26]. The NRF Index arrives at a continuous composite nutritional score by subtracting the LIM score (expressed per 100 kcal instead of per 100 g) from the density score of nine beneficial nutrients (i.e., protein, fiber, vitamins A, C, and E, iron, calcium, potassium, and magnesium) per 100 kcal of a food. Higher scores indicate higher nutritional quality [30].

### Data analysis

Identical but separate analyses were performed for each list.

#### Quality control

To ensure the evaluators displayed caution and honesty when completing the survey, we performed a quality control test using five foods per list for which the NOVA group should have been obvious. From the list of marketed foods, we selected beef bourguignon and potatoes, fruit dairy dessert, Chinese fried rice, toasted bread with fruit chips (expected assignment of NOVA4 for all four foods), and plain yogurt (expected assignment of NOVA1) (see Supplementary Table 1). From the list of generic foods, we selected apple, lettuce, egg (expected assignment of NOVA1 for all three), butter (expected assignment of NOVA2), and soda (expected assignment of NOVA4). When evaluators arrived at an erroneous assignment for more than one test food, their data were excluded from the analysis.

We also excluded any data from evaluators who failed to assess all the foods on the list(s), which allowed us to better ensure that evaluators were committed to their task, and to limit confusion that may arise from statistical analyses based on different sample sizes of foods and/or evaluators.

#### Description of the data

The NOVA system does not provide “gold-standard references” to which the evaluators’ assignments could be compared. To describe the raw data obtained (i.e., the assignments), we calculated, for each food, the percentage of assignments in each of the four NOVA groups and, for each list, the number of foods assigned to one, two, three, or four different NOVA groups.

#### NOVA assignment patterns

Each evaluator assessed each food, assigning it to a NOVA group. Using these data, we obtained a frequency table for each food list (Supplementary Tables 1 and 2) on which a correspondence analysis (CA) was performed. The spatial pattern of these NOVA assignments was then graphically represented. Furthermore, for each food list, the degree of association between the foods and the NOVA assignments was quantified using Cramer’s V. The value of this coefficient varies from 0 to 1, where 1 signified that the NOVA assignments were 100% consistent for each food (i.e., all the evaluators assigned a given food to the same group).

#### Consistency among evaluators

We estimated Fleiss’ κ to quantify the degree of agreement in the evaluators’ NOVA assignments; we used an overall sample based on the mean of 1000 bootstrapped samples [31]. Fleiss’ κ can range from 0 to 1, where 1 indicates full agreement. Each bootstrapped sample was stratified by professional background. There were at least 10 evaluators representing each type of professional background, leading to a bootstrapped sample size of 70 evaluators. This strategy was chosen to detect whether experts with similar professional expertise might be evaluators with higher concordance.

#### Food clusters arising from NOVA assignments

We performed hierarchical clustering on principal components (HCPC) to identify clusters of foods that displayed similar distributions of assignments among the four NOVA groups. If each food had been assigned to the same NOVA group by all the evaluators, the HCPC analysis would have yielded four clusters, each 100% composed of foods exhibiting the same NOVA assignment. Consequently, the clusters of foods reflected differences in assignment distributions, helping us identify similar and dissimilar distribution patterns. For example, HCPC could yield (i) a cluster in which most foods had been assigned to a given group or (ii) a cluster in which most foods had been assigned to three or four groups. The latter case would be a sign that evaluators were highly inconsistent in their assignments, and the foods in such clusters would merit further examination.

The clustering algorithm utilized Ward’s method [32], and the number of clusters was set to obtain the smallest amount of within-cluster variation possible. We determined the percentage of NOVA1, NOVA2, NOVA3, and NOVA4 assignments in each cluster.

#### Sensitivity analyses

We identified evaluators who produced atypical assignments based on a recent method described by Lindskou et al. [33]., which detects outliers in contingency tables. Then, excluding the data from these outlier evaluators, we performed sensitivity analyses on the Fleiss’ κ values and the HCPC results to verify the robustness of our main analyses.

#### NOVA assignments and nutritional quality

We first defined the most common assignment made by the evaluators for each food (i.e., NOVA_maj_). For instance, for a food that could have been assigned NOVA1 by 5% of evaluators, NOVA2 by 15% of evaluators, NOVA3 by 35% of evaluators and NOVA4 by 45% of evaluators, we retained that this particular food was mainly assigned NOVA4 (i.e. NOVA4_maj_). Using chi-squared tests, we explored the relationships between NOVA_maj_ categories and their Nutri-Score and SAIN,LIM classes. Then, for each NOVA_maj_ category, the distributions of the values of Nutri-Score, SAIN, LIM, NRF 9.3, and energy density were graphed using boxplots, and statistical comparisons among NOVA_maj_ categories were carried out using a non-parametric test (Kruskal-Wallis).

We performed all the statistical analyses using R (v. 4.0.2.); we employed the package Irr to assess consistency among evaluators; FactoMineR to perform the CA and the HCPC; and DeskTool to calculate the Cramer’s V values. For all statistical tests, an alpha level of 5% was used.

## Results

### Food lists and evaluators

The list of marketed foods and the list of generic foods were assessed by 196 and 202 evaluators, respectively; 144 evaluators completed both lists. A total of 62 evaluators were excluded (List of marketed foods: 37 evaluators, List of generic foods: 25 evaluators), either because they did not assess all the foods on the list (List of marketed foods: 30 evaluators, List of generic foods: 24 evaluators) or because they failed the quality control test (List of marketed foods: 7 evaluators, List of generic foods: 1 evaluator). Consequently, data from 159 evaluators (List of marketed foods) and 177 evaluators (List of generic foods) were used in the analyses. Thus, in total, 19,080 and 19,647 assignments were obtained for the 120 marketed foods and the 111 generic foods, respectively.

### Distribution of NOVA assignments for the food lists and confidence of evaluators

**Fig. 1:**
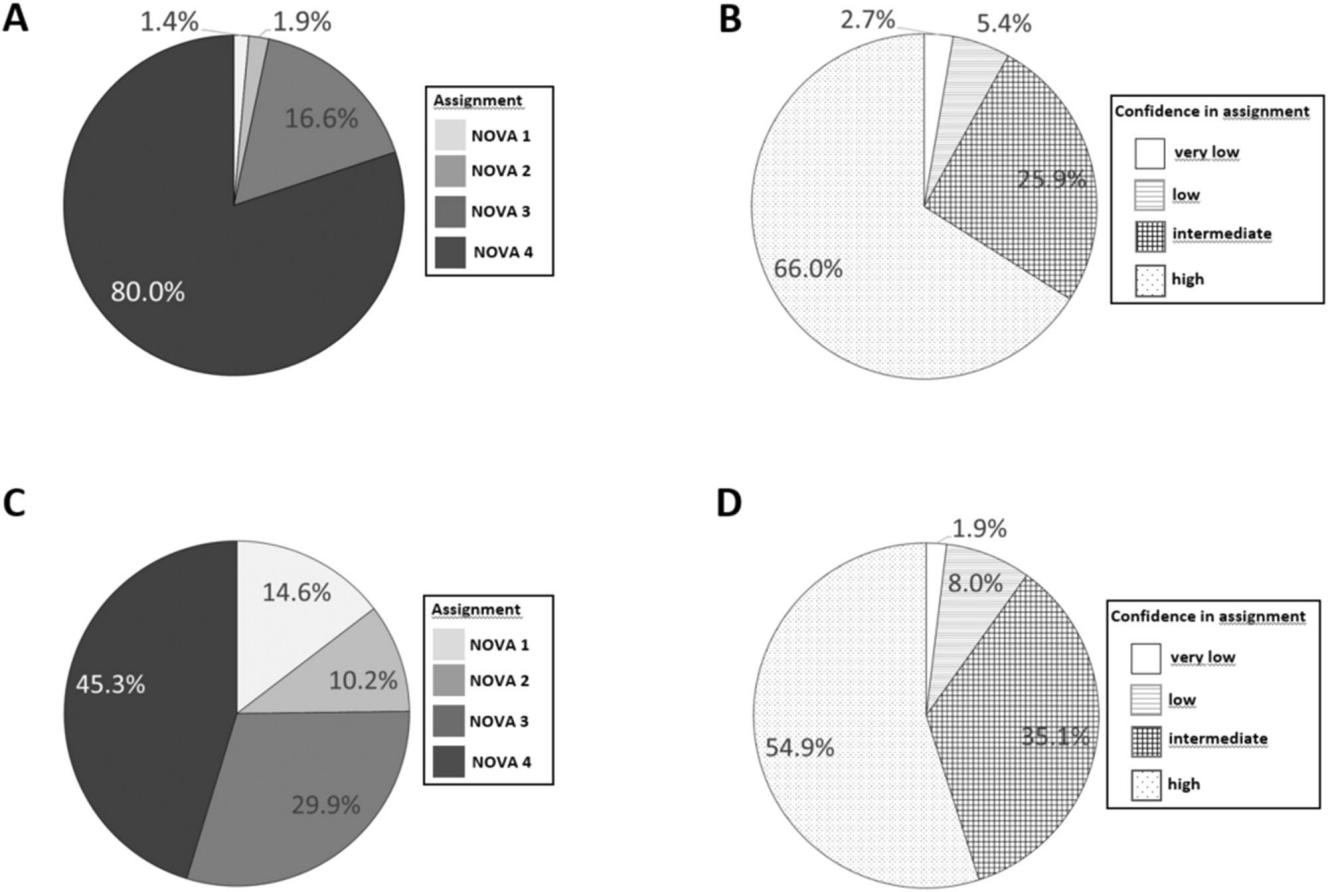
Distribution of NOVA assignments. For the marketed foods (list with ingredient information provided; **A** *N* = 19,080) and the generic foods (no ingredient information provided; **C** *N* = 19,647) as well as evaluator confidence in the assignments (**B** 19,080 marketed foods, **D** 19,647 generic foods).

Most of the marketed foods were assigned to NOVA4 (80.0% of the 19,080 assignments). The next most common assignment was NOVA3; there were only a few NOVA1 and NOVA2 assignments (Fig. 1A). Similarly, the generic foods were most frequently assigned to NOVA4 (45.3% of the 19,647 assignments); the next most common assignments were, in order of frequency, NOVA3, NOVA1, and NOVA2 (Fig. 1C). For both lists, evaluators mainly had “high” or, less commonly, “intermediate” confidence in their assignments; fewer than 10% of evaluators indicated that their level of confidence was “low” or “very low” (Fig. 1B and D).

### Relative frequency of NOVA assignments

Only three marketed foods and one generic food were assigned to the same NOVA group by all the evaluators, and most of the foods in both lists were placed in two, three, or even four NOVA groups (Table 1). In cases where foods were assigned to two or three groups, NOVA4 was usually the most common assignment (e.g., when a given food was assigned to three different groups, 83.9% of the time at least one of the groups was NOVA4). When foods were assigned to all four groups, the assignments were more evenly distributed. The NOVA assignment frequencies for the foods in both lists are in the supplementary materials (Supplementary Tables 1 and 2).

**Table 1:** Number of foods in the two lists that were assigned to one, two, three, or four NOVA groups and relative frequency of the different assignments.

	Foods (*n*)	Assignments (*n*)	% of NOVA1 assignments	% of NOVA2 assignments	% of NOVA3 assignments	% of NOVA4 assignments
*Marketed foods*						
Assigned to one group	3	477	0	0	0	100
Assigned to two groups	50	7950	0	0	4.6	95.4
Assigned to three groups	27	4293	0.03	1.5	14.6	83.9
Assigned to four groups	40	6360	6.7	7.4	44.8	41.0
*Generic foods*						
Assigned to one group	1	177	100	0	0	0
Assigned to two groups	9	1593	22.1	0.06	6.3	71.5
Assigned to three groups	17	3009	9.8	3.8	17.9	68.5
Assigned to four groups	84	14,868	13.8	12.7	35.2	38.3

**Fig. 2:**
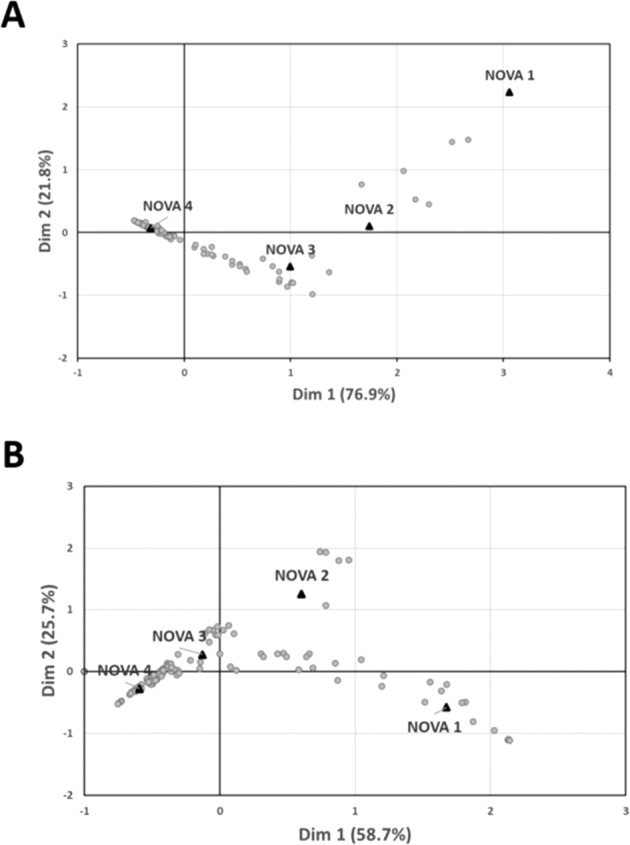
Distribution of NOVA assignments across foods based on the correspondence analysis results for each list. **A** Marketed foods (list with ingredient information provided), **B** generic foods (no ingredient information provided). Each dot corresponds to a single food, which is positioned closer to or farther from one of the four NOVA groups (triangles) based on assignment frequencies. Thus, if evaluators were to be in 100% agreement regarding a food assignment, the food dot would occur on top of the triangle representing the relevant NOVA group.

We examined the pattern of NOVA assignments that arose from the CA results for each list (Fig. 2). Although the foods tended to form distinct assignment-based clouds, we nonetheless observed pronounced inconsistency in the evaluators’ assignments, as revealed by the Cramer’s V values, which were 0.58 and 0.59 for the marketed foods and the generic foods, respectively.

#### Consistency among evaluators

The mean values of Fleiss’ κ were 0.32 among the evaluators who assessed the marketed foods and 0.34 among the evaluators who assessed the generic foods. Professional background did not affect consistency among evaluators, which was low for both lists (Table 2). When outliers were filtered out (List of marketed foods: 5 evaluators removed, List of generic foods: 12 evaluators removed), the mean values of Fleiss’ κ improved slightly, by a maximum of 0.03 (data not shown).

**Table 2:** NOVA assignment consistency among evaluators overall and based on professional background.

	Marketed foods			Generic foods		
	Eval. (*N*)	Mean *κ*	95% CI	Eval. (*N*)	Mean κ	95% CI
All	159	0.32	0.27–0.37	177	0.34	0.30–0.37
Health professionals	23	0.33	0.19–0.51	25	0.37	0.29–0.45
Researchers—food technology	19	0.31	0.21–0.43	27	0.28	0.18–0.40
Researchers—human nutrition	68	0.32	0.23–0.41	69	0.37	0.32–0.43
Industry R&D professionals	49	0.35	0.27–0.43	56	0.34	0.29–0.41

The mean Fleiss’ κ values and 95% confidence intervals (CIs) were obtained from 1000 bootstrapped samples.

*Eval* evaluators, *κ* Fleiss’ κ.

Three clusters (called T, U, and V) were formed among the marketed foods, and four clusters (called W, X, Y, and Z) were formed among the generic foods (Table 3). To illustrate cluster composition, cluster W contained 65 foods associated with 11,505 assignments (i.e., *N* = 65 food products * 177 evaluators); the foods had mostly been assigned to NOVA4 (69%), although 26%, 4%, and 1% had been assigned to NOVA3, NOVA1, and NOVA2, respectively.

**Table 3:** Food clusters that arose from the NOVA assignments.

Cluster	Foods (*n*)	Assignments (*n*)	% of NOVA1 assignments	% of NOVA2 assignments	% of NOVA3 assignments	% of NOVA4 assignments
*Marketed foods (N* *=* *120)*						
T	90	14,310	0.11	0.65	8.55	90.7
U	24	3816	1.39	4.77	40.3	53.6
V	6	954	23.3	14.5	48.0	14.3
*Generic foods (N* *=* *111)*						
W	65	11,505	1.01	3.7	25.8	69.5
X	28	4956	16.6	13.4	53.2	16.8
Y	5	885	13.3	74.5	10.1	2.15
Z	13	2301	78.9	11.1	7.78	2.26

Within the marketed food clusters, cluster T contained most of the list’s foods and was very homogeneous (90.7% of assignments were NOVA4). There were 30 foods total across the two other clusters (i.e., U = 24, V = 6), which displayed more heterogeneous assignment patterns. In cluster U, most foods had been assigned to NOVA3 or NOVA4; in cluster V, most foods had been placed in NOVA3, although NOVA1, NOVA2, and NOVA4 were also represented.

Within the generic food clusters, clusters Y and Z contained foods that had largely been assigned to NOVA2 and NOVA1, respectively. Cluster W contained 65 foods that had mostly been assigned to NOVA4 (69%), followed by NOVA3 (26%). Finally, cluster X contained 28 foods with more heterogeneous assignments.

For the generic foods, a single NOVA assignment predominated within the clusters W, Y, and Z. For example, 78.9% of the foods in cluster Z had been assigned to NOVA1. In contrast, in cluster X, foods had been assigned to all four NOVA groups, although NOVA3 assignments were slightly more common (53%). When the data from the outlier evaluators were removed, cluster composition remained the same (data not shown).

When we examined the foods with the most inconsistent assignments (Supplementary Table 3), we observed certain patterns. The 30 foods in clusters U and V were mainly plain and unsweetened fresh dairy products (all the foods in cluster V) and breads/bread-like foods (58% of the foods in cluster U). In the highly heterogeneous cluster X, 8 of the 28 total foods were yogurts, cheeses, or fromage frais (out of the 20 dairy products in the list), and 7 were breads (out of the 9 breads in the list) (Supplementary Table 3 and Supplementary Table 2). When labeled as “commercial”, orange juice was found in this cluster: 16% of assignments were NOVA1, 11% were NOVA2, 34.5% were NOVA3, and 38.5% were NOVA4. Fresh orange juice displayed a completely different distribution of assignments (NOVA1 = 66%, NOVA2 = 21%, NOVA3 = 12.4%, and NOVA4 = 0.6%) (Supplementary Table 2).

### NOVA_maj_ assignments and nutritional quality

**Fig. 3:**
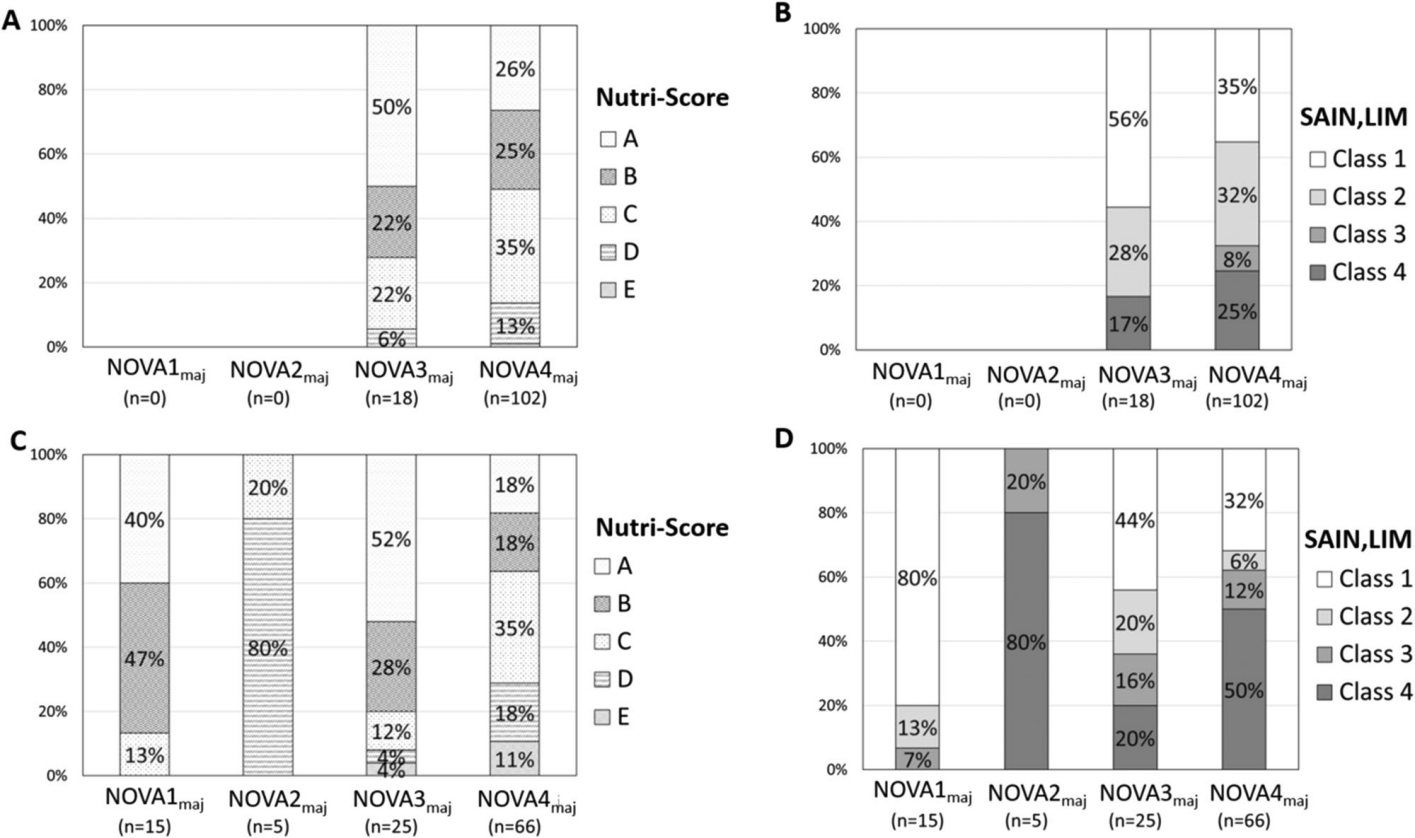
Distribution (in %) of the NOVA_maj_ assignments based on nutritional quality. Distribution as defined by Nutri-Score (**A**, **C**) and SAIN-LIM (**B**, **D**) values for marketed foods (**A,** **B**) and generic foods (**C,** **D**). Nutritional quality decreases from panels **A** to **E** (Nutri-Score) and from Class 1 to Class 4 (SAIN-LIM).

When the analyses were performed according to the Nutri-Score and the SAIN,LIM classes (Fig. 3), NOVA4_maj_ marketed foods were distributed in all classes of nutrient profiles, including the healthier ones (e.g., 26% and 35% of NOVA4_*maj*_ foods were in class A of the Nutri-Score and in class 1 of the SAIN,LIM, respectively). For the generic foods, NOVA4_maj_ foods were distributed in all classes of nutrient profiles, including the healthier ones (e.g., 18% and 32% of NOVA4_maj_ foods were in class A of the Nutri-Score and in class 1 of the SAIN,LIM, respectively) (Fig. 3).

NOVA3_maj_ marketed foods were of higher nutritional quality than NOVA4_maj_ marketed foods based on the Nutri-Score, SAIN, and LIM values; no such difference was seen with the energy density or NRF 9.3 values (Supplementary Fig. 2A). For the generic foods, NOVA1_maj_ foods were of higher nutritional quality than NOVA3_maj_ foods; in turn, NOVA3_maj_ foods were of higher nutritional quality than NOVA4_maj_ foods. NOVA2_maj_ foods always displayed the worst nutritional quality (Supplementary Fig. 2B).

## Discussion

In this study, we explored the robustness and functionality of the NOVA classification system by asking food and nutrition specialists to implement the system as intended by its creators [9]. We had them assess a list of marketed foods and a list of generic foods commonly consumed in France. The most striking result was that evaluators were inconsistent in their assignments, regardless of professional background; the mean values of Fleiss’ κ never exceeded 0.34. Many foods were not consistently assigned to the same NOVA group. In particular, the HCPC analysis indicated that assignments were highly heterogeneous for 30 marketed foods (25% of the total) and 28 generic foods (25% of the total). Finally, we found that an appreciable percentage of the foods commonly considered to be ultra-processed (NOVA4_maj_) were of acceptable nutritional quality.

To date, only one previous study has addressed similar questions. It found that reliability between two evaluators was lower with the NOVA system than with two other similar classification systems [16]; it highlighted that the risk of misclassification was higher when using NOVA probably because its four groups are not clearly defined. We found support for this idea using a much larger number of evaluators.

Surprisingly, providing detailed ingredient information did not improve evaluator consistency nor did it affect evaluator confidence levels. The latter were high or very high for most of the assignments, whether or not ingredient information was present. This result suggests that evaluators relied on their own knowledge or subjective feelings about the foods when making their assignments.

Some foods had a wider range of assignments. For instance, plain unsweetened dairy products were assigned to all four NOVA groups (cluster V; Table 3). This result may be tied to ambiguity in NOVA criteria [9]. On the one hand, “yogurt with no added sugar or artificial sweeteners” is specifically cited as an example of a NOVA1 food. On the other hand, it is clearly stated that non-alcoholic fermentation, the process by which yogurt is made (i.e., lactic fermentation), is characteristic of NOVA3 foods. It is further mentioned that “substances […], such as casein, lactose, whey”—ingredients often present in yogurt—are “only found in ultra-processed products,” meaning NOVA4 foods [9]. It was equally hard to determine whether other foods belonged in NOVA3 or NOVA4 (e.g., see cluster U; Table 3). One source of uncertainty is the indication that ultra-processed foods (NOVA4) “are industrial formulations, typically with five or more and usually many ingredients,” which may have led some evaluators to assign foods with long ingredient lists to NOVA4, even if they did not contain ingredients typical of NOVA4, such as “substances not commonly used in culinary preparations.” This reference to culinary versus industrial processes for preparing foods may also have led to misunderstanding: for example, corn starch is not necessarily a common ingredient in French households, which may have resulted in corn starch-containing foods being assigned to NOVA4. However, corn starch is also cited as an example of a NOVA2 item [9], and its presence may thus have led to NOVA3 assignments as well. Finally, uncertainty can stem from the processing procedure. For example, popcorn cakes may be treated as a food that has undergone extrusion cooking, leading some evaluators to arrive at a NOVA4 assignment. However, other evaluators may have opted for NOVA3 instead, given the food’s simple ingredient list. Overall, NOVA appears to be overly reliant on non-hierarchical criteria that cannot be applied rigorously and systematically in the absence of an unambiguous decision tree.

In the case of the generic foods, evaluators had no information about ingredients. When considering the 28 generic mixed dishes, two-thirds of the assignments were NOVA4, likely because evaluators assumed the foods had been industrially produced. Other evaluators arrived at assignments of NOVA3, likely assuming the foods were homemade. Interestingly, we observed the same ambiguity surrounding the generic yogurts and fromage frais as for the marketed dairy foods, underscoring the contradictory criteria of NOVA regarding these products. We also discovered that foods perceived to be industrial in nature were more likely to be assigned to NOVA4. For example, evaluators largely placed commercial orange juice in NOVA4, even though “fresh, squeezed, chilled, frozen, or dried fruits” are mentioned among the NOVA1 examples [9]. Similarly, 70% of evaluators classified coffee as NOVA1. Coffee torrefaction is often industrial in nature, and food technology specialists view it as a high-impact process whose elevated temperatures lead to high acrylamide levels [34]. However, the NOVA system surprisingly allows torrefaction in association with NOVA1 foods because it is a traditional processing method. Similarly, the evaluators seem to have based their assignments of coffee on cultural rather than scientific knowledge, likely because this beverage is familiar and frequently consumed in France.

The definition of levels of food processing, as proposed by the NOVA classification, is complex and multidimensional [17]. It does not really reflect the intensity of the processes used, but is a mix of technological considerations based more on socio-cultural aspects than on physical-chemical ones occurring during food processing. Furthermore, NOVA criteria associates such so-called technological dimensions with formulation considerations, such as the use of some specific ingredients, or the number of total ingredients involved in the recipe. Separating the level of thermo-mechanical energy undergone by the raw material from the formulation of the food (and in particular from the addition of additives) could be a way for building a robust indicator of the level of food processing. Such an indicator of food processing could help for better understanding if the links observed between ultra-processed food consumption and health are mainly due to the food structure or to the food composition (specific ingredients and additives). For this, the construction of an analysis grid by major categories of unit operations required for food processing would be necessary. Understanding the links between the consumption of highly processed foods and health must necessarily integrate very interdisciplinary skills including food process engineering, food sciences, nutrition, and nutritional epidemiology.

Several studies have found that people who consume more ultra-processed foods (i.e., as defined by NOVA) have higher sugar and lower fiber intake; few differences exist from low ultra-processed foods consumers in their sodium, total fat, and saturated fat intake, but consumption of vitamins and minerals may vary [35–41]. Here, we found that foods most commonly assigned to NOVA4 (i.e., NOVA4_maj_ foods) could vary substantially in their nutrient profiles, possibly in relation to the large heterogeneity of their composition. Thus, diet quality is more likely to be determined by specific consumer choices from among NOVA4 foods than by a food’s assignment to NOVA4 in and of itself. Confusing messages may arise from front-of pack labeling, such as when products with a NOVA4 label, signifying their ultra-processed nature, would also bear a label conveying their good nutritional quality (e.g., Nutri-Score “A”).

This study has limitations. First, regarding the choice of the food lists, there were only three food categories for the marketed foods and different results might have been obtained if other categories had been used. Our goal was to balance survey duration with reasonable food representation, allowing us to explore potentially conflicting NOVA assignments. Representativeness was greater for the generic foods, which were taken from the results of a population-level dietary survey. Second, while all the evaluators were French, they were specialists in human nutrition and/or food technology; their expertise should thus have served to overcome potential cultural bias and ensure the validity of our data set.

Third, the survey’s structure did not allow evaluators to modify earlier assignments, meaning they could not apply any understanding they developed as they advanced. Hypothetically, the ability to modify assignments could have slightly improved evaluator consistency, as could have allowing real-life discussions among evaluators. Finally, the representativeness of evaluators is also questionable.

However, our study also had several strengths. First, we obtained data from more than 150 evaluators for a total of 231 foods, yielding a much larger data set and more powerful statistical analysis than in previous studies [16, 42]. Second, extreme caution was taken to avoid influencing the evaluators in any way. Our online survey interface facilitated participation and created controlled study conditions. All evaluators were given the exact same information about NOVA, taken from the original publications. The survey set-up provided easy access to the description of the NOVA system, accessible in full detail or as a summary. The food lists and blocks appeared in a randomized order to avoid habituation bias.

Third, the vast majority of evaluators appeared to take the assignment process seriously. Just 7 evaluators (less than 2% of the total) failed to do so. The sensitivity analyses confirmed the robustness of our findings. Consequently, we feel confident that we obtained data from individuals who assessed the foods as best they could, given current NOVA criteria. All the evaluators were specialists in human nutrition and/or food technology and thus represent the body of individuals who may have to use NOVA in their professional lives.

NOVA “multidimensionnel”

## Conclusions

Overall, our results suggest improvements should be made to the NOVA classification system to enhance assignment consistency. Indeed, we observed that a large percentage of the food assignments were discordant, regardless of whether ingredient information was provided. This finding raises questions about how functional NOVA is in its current form. It should also spur reflection on the reliability of conclusions from epidemiological studies that use NOVA as well as on NOVA’s ability to guide public health policy or provide useful information to consumers. While the concept of ultra-processed foods has certainly entered the consumer consciousness, our results indicate that NOVA criteria do not currently allow foods to be unequivocally defined as ultra-processed.

## Supplementary information

Supplementary Figure 1

Supplementary Figure 2

Supplementary Table 1

Supplementary Table 2

Supplementary Table 3

Online Supplementary Material 1

Online Supplementary Material 2

Online Supplementary Material 3

Online Supplementary Material 4
