# Supplementary material for: Ultra-processed foods: how functional is the NOVA system?
Source: Eur J Clin Nutr. 2022 Mar 21;76(9):1245–53. doi: 10.1038/s41430-022-01099-1 (PMC9436773; doi:10.1038/s41430-022-01099-1)
Supplement: Supplementary file 1 — Supplementary Figure 1 [file 41430_2022_1099_MOESM1_ESM.docx]

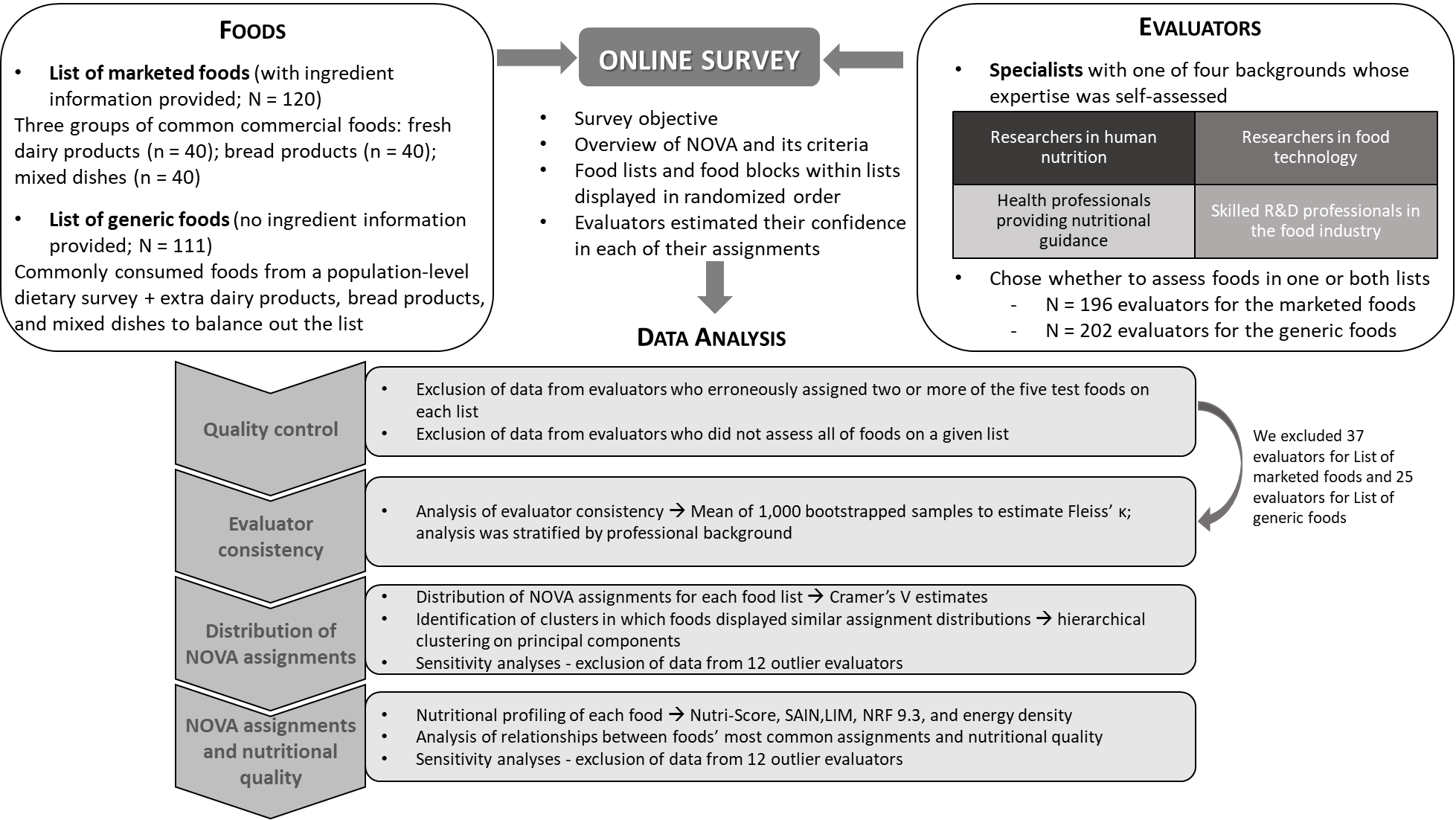


**Supplementary Figure 1:** Schematic of the study’s experimental design. Abbreviations: NRF, Nutrient Rich Food Index.
