# Supplementary material for: Ultra-processed foods: how functional is the NOVA system?
Source: Eur J Clin Nutr. 2022 Mar 21;76(9):1245–53. doi: 10.1038/s41430-022-01099-1 (PMC9436773; doi:10.1038/s41430-022-01099-1)
Supplement: Supplementary file 2 — Supplementary Figure 2 [file 41430_2022_1099_MOESM2_ESM.docx]

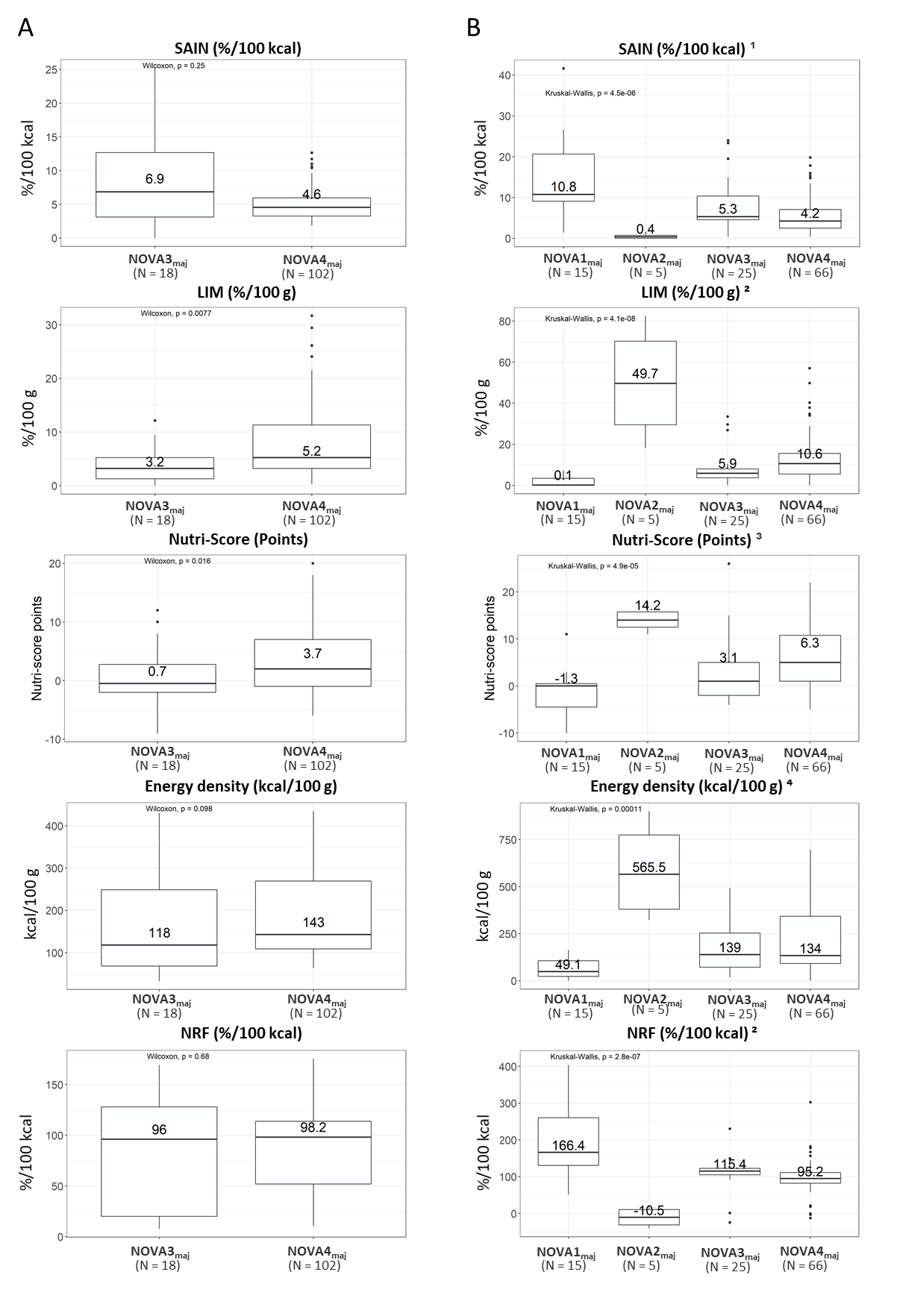


**Supplementary Figure 2.** Different nutritional profiles of the foods based on the NOVA group to which they were most commonly assigned (NOVA_maj_). Higher SAIN and NRF 9.3 values indicate higher nutritional quality; higher LIM, Nutri-Score, and energy density values indicate lower nutritional quality. (A) Marketed foods (List with ingredient information provided). (B) Generic foods (no ingredient information provided). N indicates the number of foods in each NOVA_maj_.

^1^Results for all the paired comparisons were significantly different, except for the comparison between NOVA3_maj_ and NOVA4_maj_ (p = 0.055 for SAIN, p = 0.52 for energy density)

^2^Results for all the paired comparisons were significantly different

^3^Results for all the paired comparisons were significantly different, except between NOVA1_maj_ and NOVA3_maj_ (p = 0.077)
