# Supplementary material for: Ultra-processed foods: how functional is the NOVA system?
Source: Eur J Clin Nutr. 2022 Mar 21;76(9):1245–53. doi: 10.1038/s41430-022-01099-1 (PMC9436773; doi:10.1038/s41430-022-01099-1)
Supplement: Supplementary file 5 — Supplementary Table 3 [file 41430_2022_1099_MOESM5_ESM.docx]

**Supplementary Table 3**. Marketed foods in clusters U and V: food name, ingredients, and percentage of NOVA assignments (N = 159 evaluators).

| **Food** | **Ingredients** | **% of NOVA1 assignments** | | **% of NOVA2 assignments** | | **% of NOVA3 assignments** | | **% of NOVA4 assignments** | |
| --- | --- | --- | --- | --- | --- | --- | --- | --- | --- |
| **Cluster U** |  | |  | |  | |  | |  |
| Creamy yogurt with fruit | whole milk yogurt; 20% cream; 8.5% sugar; 8% strawberry (strawberry, raspberry, or peach and passion fruit; fruit juice obtained from concentrate); 2.5% glucose-fructose syrup; concentrated red beet juice; natural aromas | | 1.3 | | 6.2 | | 63.8 | | 28.7 |
| Rice pudding | 82% whole milk; 8.6% rice; sugar; milk protein | | 1.3 | | 10.6 | | 47.5 | | 40.6 |
| Plain *fromage frais* B | pasteurized skimmed milk; pasteurized cream; lactic ferments; rennet | | 6.3 | | 11.2 | | 53.1 | | 29.4 |
| Sweetened yogurt with fruit A | partially skimmed milk; 10.2% strawberry; 9% sugar; milk protein; glucose-fructose syrup; modified corn starch; thickener (guar gum); acidifiers (citric acid, calcium lactate); aromas; coloring (carmine); fruit preservative (potassium sorbate); lactic ferments | | 3.1 | | 4.4 | | 47.5 | | 45.0 |
| Pocket bread A | wheat flour; water; salt; yeast; wheat gluten; colza oil; preservative (calcium propionate); sugar; malt wheat flour; sesame seeds | | 0.0 | | 3.8 | | 33.1 | | 63.1 |
| Pocket bread B | wheat flour; water; yeast; salt; corn dextrose | | 0.0 | | 6.3 | | 38.1 | | 55.6 |
| Flatbread crackers | 52% whole wheat flour; 46% wheat flour; sugar; canola oil; skimmed milk powder; salt | | 0.6 | | 6.9 | | 57.5 | | 35.0 |
| Milk bread rolls A | wheat flour; water; brown sugar; colza oil; eggs; sourdough obtained from rye and malted wheat flour; 2% skimmed milk powder; yeast; salt; vanilla extract; natural flavor; guar fiber | | 0.6 | | 1.9 | | 28.8 | | 68.7 |
| Popcorn cakes | 99.6% corn; sea salt; sesame; spelled | | 3.8 | | 16.2 | | 60.0 | | 20.0 |
| Precooked baguettes | wheat flour; water; yeast; salt; alcohol; broad bean flour; antioxidant (E300) | | 0.0 | | 5.0 | | 45.6 | | 49.4 |
| Precooked bread | wheat flour; water; yeast; salt; broad bean flour; antioxidant: ascorbic acid | | 1.2 | | 6.9 | | 62.5 | | 29.4 |
| Zwieback toast | wheat flour; whole wheat flour; gluten; yeast; sunflower vegetable oil; flour treatment agent (ascorbic acid) | | 0.0 | | 3.8 | | 41.2 | | 55.0 |
| Melba Toast | 89% wheat flour; palm oil; salt; sugar; yeast; dextrose; malt wheat flour; emulsifier (E472e); flour treatment agents (E920, E300) | | 1.3 | | 2.5 | | 66.2 | | 30.0 |
| Melba Toast A | wheat flour; sugar; vegetable fats; yeast; salt | | 0.6 | | 2.5 | | 45.0 | | 51.9 |
| Melba Toast B | 92% wheat flour; sugar; vegetable fats (palm oil, canola oil); yeast; salt; flour treatment agent (ascorbic acid) | | 0.0 | | 3,8 | | 48.7 | | 47.5 |
| Whole wheat Melba Toast A | 50% whole wheat flour; wheat flour; vegetable fats (palm oil, canola oil); sugar; yeast; salt; gluten; flour treatment agent (ascorbic acid) | | 0.0 | | 2.5 | | 31.9 | | 65.6 |
| Whole wheat Melba Toast B | wheat flour; 30% whole wheat flour; colza oil; 4.2% brown flax seeds; yeast; salt; flour treatment agent (ascorbic acid) | | 0.6 | | 8.1 | | 71.9 | | 19.4 |
| Whole wheat zwieback toast | 89% whole wheat flour; sunflower oil; sugar; yeast; salt; wheat gluten; flour treatment agent (ascorbic acid) | | 0.6 | | 3.8 | | 43.1 | | 52.5 |
| Cooked green beans B | 70% green beans; 10% cooked smoked bacon (pork belly, salt, dextrose, smoke flavor, citric acid, sodium erythorbate, sodium nitrite); 8% pre-fried onions (onions, canola oil); butter; salt; parsley; garlic; sugar; chives; potato starch; natural flavor; processed starch; pepper | | 1.3 | | 8.7 | | 53.1 | | 36.9 |
| Cooked lentils | 55% lentils; cooked sauce (broth prepared the old-fashioned way from rinds and 3% goose fat; tomato paste; spices; aromatics; salt; wheat dextrose; natural flavors; chicken fat); carrots; onions | | 0.6 | | 5.6 | | 26.9 | | 66.9 |
| Salted pork and lentils | cooked sauce (water, duck fat, salt, tomato paste, garlic, pepper); 32% cured pork knuckle (water, salt, pepper); 18% green lentils; 3% smoked bacon (water, salt); 3% baby onions; 2% carrots | | 1.3 | | 5.0 | | 60.0 | | 33.7 |
| Poultry with chestnuts and mushrooms | 79% cured semi-boneless cockerel (95.5% semi-boneless cockerel, water, wheat glucose syrup, salt); 21% stuffing (64.6% pork, 9.2% chestnuts, 9.2% dehydrated porcini mushrooms [dehydrated porcini mushrooms, water]; cooked onions [onions, non-hydrogenated sunflower oil, salt], water, parsley, salt, pepper) | | 0.0 | | 1.9 | | 31.2 | | 66.9 |
| Ratatouille | 35.5% tomatoes; 34% cubed zucchini; 6% eggplant cubes; tomato puree; 5% cubed onions; 4% diced red peppers; sunflower oil; 2% cubed green peppers; cane sugar; thickener (wheat starch); Guerande salt; natural aromas; acidifier (citric acid) | | 0.6 | | 1.3 | | 33.7 | | 64.4 |
| Salmon and vegetable risotto | 52% risotto cooked with parmesan (45% cooked rice [water, rice], white wine, shallots, 1% parmesan, salt, olive oil); 48% salmon and leeks with cream (15.5% Atlantic salmon, water, 9% *crème fraîche*, 7.5% leeks, Emmental cheese, fennel, mozzarella, fish stock [fish meat, fish juice, salt], corn starch, garlic, lemon juice, spice extracts, cumin) | | 0.6 | | 3.8 | | 36.9 | | 58.7 |
| **Cluster V** |  | |  | |  | |  | |  |
| Plain fermented milk | whole milk; lactose and milk protein; lactic ferments; *bifidobacteria* | | 20.0 | | 13.1 | | 38.1 | | 28.8 |
| Plain *fromage frais* A | skimmed milk; cream; lactic ferments; preservatives (E203) | | 34.4 | | 15.6 | | 48.1 | | 1.9 |
| Plain *fromage frais* C | pasteurized skimmed milk; pasteurized cream; lactic ferments | | 21.3 | | 15.6 | | 56.9 | | 6.2 |
| Plain *fromage frais* D | pasteurized skimmed milk; lactic ferments | | 21.9 | | 16.3 | | 61.2 | | 0.6 |
| Plain yogurt A | whole milk 94%; skim milk powder; lactic ferments | | 25.0 | | 16.3 | | 41.2 | | 17.5 |
| Plain yogurt B | whole milk 98%; milk protein; lactic ferments | | 33.1 | | 14.4 | | 45.6 | | 6.9 |
