# Supplementary material for: Ultra-processed foods: how functional is the NOVA system?
Source: Eur J Clin Nutr. 2022 Mar 21;76(9):1245–53. doi: 10.1038/s41430-022-01099-1 (PMC9436773; doi:10.1038/s41430-022-01099-1)
Supplement: Supplementary file 6 — Online Supplementary Material 1 [file 41430_2022_1099_MOESM6_ESM.pdf]

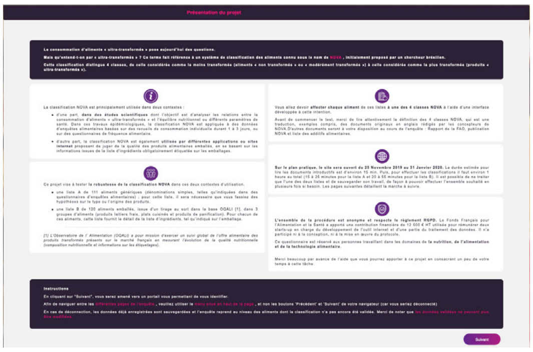

a

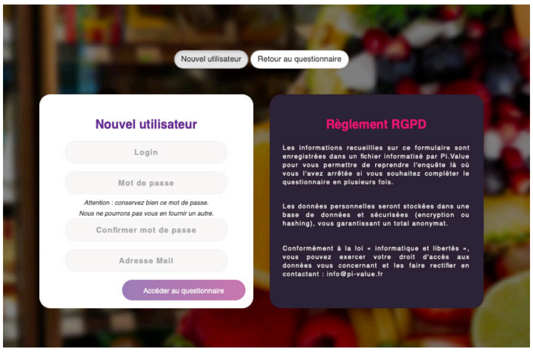

b

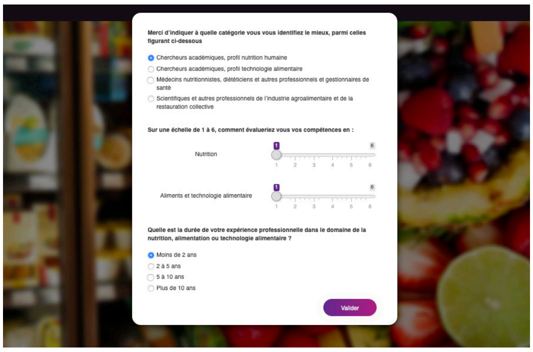

c

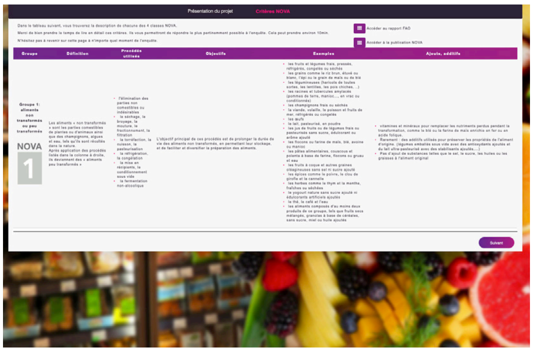

d

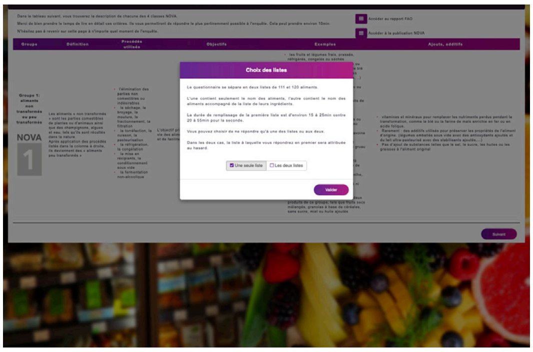

e

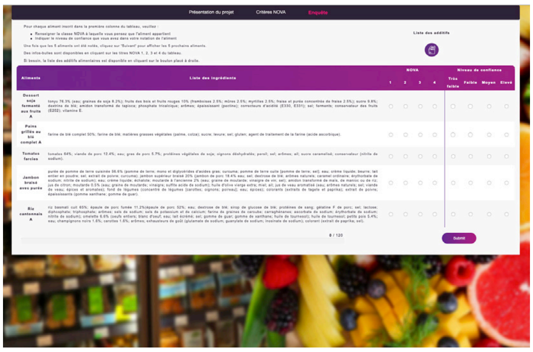

f

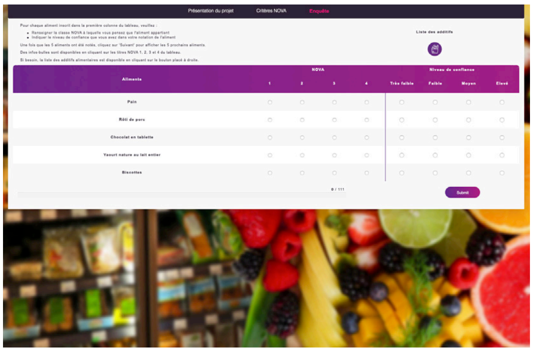

g

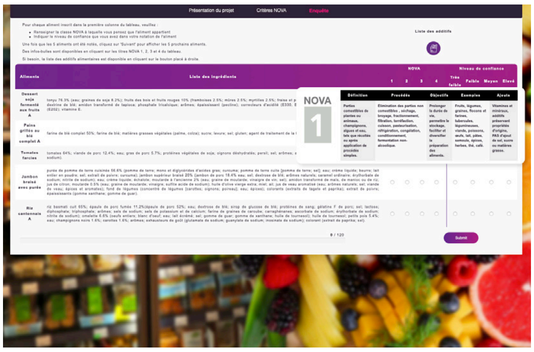

h

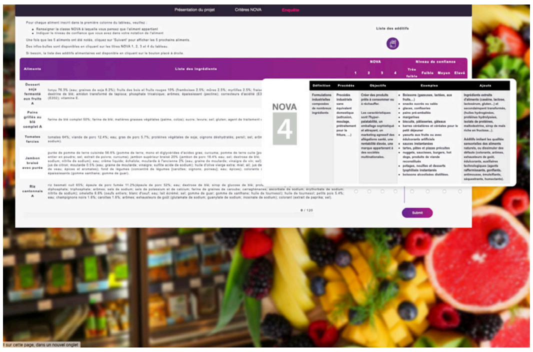

i

Online Supplementary Material 1: Screenshots of the on-line survey: (a) Survey description (for more details see Online Supplementary Material 2); (b) evaluator registration page; (c) evaluator professional background page; (d) French translation of the Nova criteria (based on Monteiro *et al.*, 2016*; for more details see Online Supplementary Material 4); (e) page where the evaluators indicated their decision to assess one or both lists (f): examples of marketed foods (List 1; ingredient information provided); (g) examples of generic foods (List 2; no ingredient information provided); (h and i) « help » pop-up windows on each page, showing criteria for NOVA1 (h) or NOVA4 (i) (for more details, see Online Supplementary Material 3).

* Monteiro CA, Cannon G, Levy R, Moubarac J-C, *et al.*. NOVA. The star shines bright. World Nutrition 2016; 7:28-38.
