# Supplementary material for: Ultra-processed foods: how functional is the NOVA system?
Source: Eur J Clin Nutr. 2022 Mar 21;76(9):1245–53. doi: 10.1038/s41430-022-01099-1 (PMC9436773; doi:10.1038/s41430-022-01099-1)
Supplement: Supplementary file 7 — Online Supplementary Material 2 [file 41430_2022_1099_MOESM7_ESM.pdf]

Online Supplementary Material 2: Introduction to the survey (English translation (left column) from the French text read by the evaluators using the online application (right column)

Currently, concerns exist about the increasing consumption of ultra-processed foods. However, what is the definition of an «ultra-processed food»? This term comes from the NOVA classification system, initially described by Brazilian researchers. In this system, there are four food groups, ranging from the least processed foods («unprocessed» or «moderately processed») to the most processed foods («ultra-processed»).

NOVA classification is mainly used in two contexts: (i) in scientific studies, where the objective is to analyze relationships between the consumption of «ultra-processed» foods and nutritional balance and/or various health parameters; in these epidemiological studies, NOVA classification is applied to data from food surveys (e.g., food consumption by individuals over 1–3 days) or from food frequency questionnaires and (ii) in the context of different applications or websites, whose stated purpose is to assess the quality of commercial food products based on their mandatory list of ingredients.

We are a small group of scientists who wish to test the robustness of the NOVA classification system in these two contexts of use. To this end, we have developed two lists of foods: (i) one contains 111 generic foods (simple names, like those that appear on food frequency questionnaires) and (ii) the other contains 120 marketed foods, which were randomly chosen from the OQALI database (1) and which represent three food groups (fresh dairy products, ready-to-eat meals, and bread products). The latter list provides detailed information on the ingredients in each food (i.e., as indicated on food packaging).

We are asking you to assign each food in these lists to one of the four NOVA groups using an interface we have developed for this purpose. Before starting the survey, we ask that you carefully read the definitions of the four NOVA groups (which we have translated from the original English-language publications written by the system's creators). For practical reasons, you will not be able to change any of your assessments once they have already been saved, so it is important that you understand all the components and criteria before you start.

On a practical level, the survey will be available from 27.11.2019 to 08.02.2020. We estimate that it will take you approximately 2 hours in total to complete the assessments (1 hour for each list). It is possible to go through only one of the two lists and to save your work, so that you can complete the assessments in more than one session if needed. The following pages describe the survey procedure. The entire process is anonymous and complies with GDPR requirements. The French Fund for Food and Health made a financial contribution of €12,500, excluding VAT, to the study, which was used to pay the two start-ups that developed the online survey interface and performed part of the data processing. This organization was not involved in study design or implementation.

We thank you in advance for your help with our research and the time you are spending on this task.

(1)The Observatoire de l'Alimentation (OQALI) monitors the overall supply of processed foods on the French market by quantifying changes in nutritional quality (i.e., via nutritional composition and labeling information).

La consommation d'aliments « ultra-transformés » pose aujourd'hui des questions. Mais qu'entend-t-on par « ultra-transformés » ? Ce terme fait référence à un système de classification des aliments connu sous le nom de NOVA, initialement proposé par un chercheur brésilien. Cette classification distingue 4 classes, de celle considérée comme la moins transformée (aliments « non transformés » ou « modérément transformés ») à celle considérée comme la plus transformée (produits « ultra-transformés »).

La classification NOVA est principalement utilisée dans deux contextes : (i) d'une part, dans des études scientifiques dont l'objectif est d'analyser les relations entre la consommation d'aliments « ultra-transformés » et l'équilibre nutritionnel ou différents paramètres de santé. Dans ces travaux épidémiologiques, la classification NOVA est appliquée à des données d'enquêtes alimentaires basées sur des recueils de consommation individuelle durant 1 à 3 jours, ou sur des questionnaires de fréquence alimentaire. (ii) D'autre part, la classification NOVA est également utilisée par différentes applications ou sites internet proposant de juger de la qualité des produits alimentaires emballés, en se basant sur les informations issues de la liste d'ingrédients obligatoirement étiquetée sur les emballages.

Nous sommes un petit groupe de scientifiques qui souhaitons tester la robustesse de la classification NOVA dans ces deux contextes d'utilisation. Dans cet objectif, nous avons établi deux listes d'aliments : (i) une liste de 111 aliments génériques (dénominations simples, telles qu'indiquées dans des questionnaires de fréquence alimentaire) et (ii) une liste de 120 aliments emballés, issue d'un tirage au sort dans la base OQALI [1], dans 3 groupes d'aliments (produits laitiers frais, plats cuisinés et produits de panification). Pour chacun de ces aliments, cette liste fournit le détail de la liste d'ingrédients, tel qu'indiqué sur l'emballage

Nous vous demandons d'affecter chaque aliment de ces listes à une des 4 classes NOVA, à l'aide d'une interface que nous avons développée à cette intention. Avant de commencer le test, nous vous demandons de lire attentivement la définition des 4 classes NOVA (issue d'une traduction que nous avons réalisée à partir des documents originaux en anglais rédigés par les concepteurs de NOVA). Pour des raisons pratiques, il ne vous sera pas possible de revenir sur vos classements déjà enregistrés et il est donc important que vous ayez connaissance de tous les éléments et critères avant de commencer.

Sur le plan pratique, le site sera ouvert du 27.11.2019 au 08.02.2020. La durée estimée pour effectuer les classifications est d'environ 2 heures au total (1 heure pour chaque liste). Il est possible de ne traiter que l'une des deux listes et de sauvegarder son travail, de façon à pouvoir effectuer l'ensemble souhaité en plusieurs fois si besoin. Les pages suivantes détaillent la marche à suivre. L'ensemble de la procédure est anonyme et respecte le règlement RGPD. Le Fonds Français pour l'Alimentation et la Santé a apporté une contribution financière de 12 500 € HT utilisée pour rémunérer deux starts-up en charge du développement de cet outil internet et d'une partie du traitement des données. Il n'a participé ni à la conception, ni à la mise en œuvre du protocole.

Nous vous remercions par avance très sincèrement de l'aide que vous pourrez nous apporter en consacrant un peu de votre temps à cette tâche.

[1] L'Observatoire de l' Alimentation (OQALI) a pour mission d'exercer un suivi global de l'offre alimentaire des produits transformés présents sur le marché français en mesurant l'évolution de la qualité nutritionnelle (composition nutritionnelle et informations sur les étiquetages).
