# Supplementary material for: Ultra-processed foods: how functional is the NOVA system?
Source: Eur J Clin Nutr. 2022 Mar 21;76(9):1245–53. doi: 10.1038/s41430-022-01099-1 (PMC9436773; doi:10.1038/s41430-022-01099-1)
Supplement: Supplementary file 8 — Online Supplementary Material 3 [file 41430_2022_1099_MOESM8_ESM.pdf]

Online Supplementary Material 3 : Information contained in the « help » pop-up windows that appeared upon request, providing a reminder of the criteria for each NOVA group. This is an English translation of the French text that was available to the evaluators.

	Definition	Process	Aim	Examples	Adds
NOVA 1	Edible parts of plants or of animals, and also fungi, algae and water, after separation from nature or after undergoing simple processes.	Removal of inedible or unwanted parts, drying, crushing, grinding, fractioning, filtering, roasting, boiling, pasteurisation, refrigeration, freezing, conditioning, non-alcoholic fermentation.	Extending life of unprocessed food, allowing their storage, facilitating and diversifying food preparation.	Fruits, vegetables, grains, corn cob or kernel, wheat berry or grain, legumes and tubers, fungi and mushrooms, meat, poultry, fish, eggs, milk, pasta, couscous and polenta, spices, herbs, tea, coffee, plain yoghourt with no added sugar or artificial sweeteners.	Vitamins and minerals lost during processes, additives preserving original properties, no addition of salt, sugar or fat.
NOVA 2	Obtained directly from group 1 foods by simple processes.	Pressing, refining, grinding, milling, and spray drying.	Make products used in home and restaurant kitchens to prepare, season and cook group 1 foods.	Salt mined or from seawater, sugar, molasses (from cane or beet), honey, vegetables oils, butter, lard, starches, vinegar, or other alcoholic drinks.	Vitamins & minerals, additives used to preserve the product's original properties.
NOVA 3	Relatively simple products obtained by adding sugar, oil, salt or other group 2 substances to group 1 foods. Most foods have 2 or 3 ingredients.	Various preservation or cooking methods and, in the case of bread and cheese, non-alcoholic fermentation.	Increase the durability of group 1 foods, or modify or enhance their sensory qualities.	Canned or bottled vegetables, fruits, legumes, salted or sugared nuts and seeds, salted, cured or smoked meats, canned fish, fruits in syrup, cheeses and unpackaged freshly made breads, alcoholic drinks produced by fermentation of group 1 (beer, cider, wine, etc.).	Additives to preserve the original properties or to resist microbial contamination.
NOVA 4	Industrial formulations typically with 5 or more and usually many ingredients.	Industrial processes with no domestic equivalents (extrusion, molding, pre-processing for frying, etc.).	Create products ready that are ready to eat, to drink or to heat; liable to replace both unrpcessed foods that are naturally ready to consumme, such as fruits or nuts, milk and water, and fresly prepared drinks, dishes, desserts and meals. Common attributes of « ultra-processed » products are hyper- palatability, sophisticated and attractive packaging, multi-media or aggressive marketing to children and adolescents, health claims, high profitability, branding and ownership by transnational corporations.	Carbonated drinks, sweet or savoury packaged snacks; ice cream, chocolate, candies (confectionery); mass-produced packaged breads and buns; margarines ans spreads; cookies (biscuits), pastries, cakes and cakes mixes; breakfast cereal, cereals and energy bars, energy drinks, fruit yoghhourts and fruit drinks, coca drinks, meat and chicken extracts and instant sauces, infant formulas, health and slimming products, pre-prepared pies and pasta and pizza dishes, poultry and fish nuggets and sticks, sausage, burgers, hot dogs, reconstituted meat products and powdered and packaged instant soups, noodles and dessert, alcohol produced by fermentation of group 1 followed by distillation (whisky, gin, rum, vodka)	Ingredients extracted from food (casein, lactose, whey, gluten, etc.) and derived from further constituents (hydrogenated or interesterified oils, hydrolysed proteins, soy protein isolates, maltodextrin, invert sugar, high fructose corn syrup). Additives such as dyes and other colours, colours stabilisers, flavours, flavour enhancers, non-sugar sweeteners, processing aids such as carbonating, firming, bulking and anti-bulking, de-foaming, anti-caking and glazing agents, emulsifiers, sequestrants and humectants.
