# Supplementary material for: Ultra-processed foods: how functional is the NOVA system?
Source: Eur J Clin Nutr. 2022 Mar 21;76(9):1245–53. doi: 10.1038/s41430-022-01099-1 (PMC9436773; doi:10.1038/s41430-022-01099-1)
Supplement: Supplementary file 9 — Online Supplementary Material 4 [file 41430_2022_1099_MOESM9_ESM.docx]

**Online Supplementary Material 4**: English translation of the French text provided to the evaluators. This description of the NOVA criteria comes from Monteiro CA, Cannon G, Levy R, Moubarac JC, Jaime P, Martins AP *et al.* *NOVA. The star shines bright*: World Nutrition, 2016.

|  | **Definition** | **Processes used** | **Process objectives** | **Examples** | **Adds** |
| --- | --- | --- | --- | --- | --- |
| **Group 1: Unprocessed or minimally processed foods** | Unprocessed (or natural) foods are edible parts of plants or of animals and also fungi, algae, and water, after separation from nature.  After undergoing the processes in the column to the right, they become “minimally processed foods” | Removal of inedible or unwanted parts; drying, crushing, grinding, fractioning, filtering, roasting, boiling, pasteurization, refrigeration, freezing, placing in containers, vacuum packaging, or non-alcoholic fermentation. | The main purpose of the processes used is to extend the life of unprocessed foods, allowing their storage for longer use, and to include facilitating and diversifying food preparation. | Fresh, squeezed, chilled, frozen or dried fruits and vegetables; grains such as brown, parboiled or white rice, corn cob or kernel, wheat berry or grain; legumes (beans of all types, lentils, chickpeas...); starchy roots and tubers (potatoes, cassava, etc., in bulk or packaged); fungi such as fresh or dried mushrooms; meat, poultry, fish and seafood, whole or in the form of steaks, fillets and other cuts, or chilled or frozen; eggs; milk, pasteurised or powdered; fresh or pasteurised fruit or vegetable juices without added sugar, sweeteners or flavours; grits, flakes or flour made from corn, wheat, oats or cassava; pasta, couscous and polenta made with flours, flakes or grits and water; tree and ground nuts and other oil seeds without added salt or sugar; spices such as pepper, cloves and cinnamon; herbs such as thyme and mint, fresh or dried; plain yoghurt with no added sugar or artificial sweeteners added; tea, coffee and drinking water. Foods made up from two or more items in this group, such as dried mixed fruits, granola made from cereals, nuts and dried fruits with no added sugar, honey or oil. | Vitamins and minerals added generally to replace nutrients lost during processing, such as wheat or corn flour fortified with iron or folic acid.  Foods in this group may infrequently contain additives used to preserve the properties of the original food. (vacuum-packed vegetables with added antioxidants and ultra-pasteurised milk with added stabilizers, ...)  No addition of substances such as salt, sugar, oils or fats to the original food |
|  | **Definition** | **Processes used** | **Process objectives** | **Examples** | **Adds** |
| **Group 2: Processed culinary ingredients.** | Substances obtained directly from group 1 foods or form nature by processes such as those described in the column to the right | Pressing, refining, grinding, milling and spray drying | In this group, the purpose of processing is to make products used in home and restaurant kitchens to prepare, season and cook group 1 foods and to make with them varied and enjoyable hand-made dishes, soups and broths, breads, preserves, salads, drinks, desserts and other culinary preparations.  Group 2 items are rarely consumed in the absence of group 1 foods. | Salt mined or from seawater; sugar and molasses obtained from cane or beet; honey extracted from combs and syrup from maple trees; vegetable oils crushed from olives or seeds; butter and lard obtained from milk and pork; starches extracted from corn and other plants.  Products consisting of two group 2 items, such as salted butter, group 2 items with added vitamins or minerals, such as iodised salt, and vinegar made by acetic fermentation of wine or other alcoholic drinks remain in this group. | Group 2 items may contain additives used to preserve the product’s original properties. Examples are vegetable oils with added anti-oxidants, cooking salt with added anti-humectants and vinegar with added preservatives that prevent microorganisms proliferation. |
|  | **Definition** | **Processes used** | **Process objectives** | **Examples** | **Adds** |
| **Group 3.**  **Processed foods** | Relatively simple products made by adding sugar, oil, salt or other Group 2 substances to Group 1 foods. Most processed foods have two or three ingredients. | Various preservation or cooking methods and, in the case of breads and cheese, non-alcoholic fermentation. | The main purpose of the manufacture of processed foods is to increase the durability of group 1 foods, or to modify or enhance their sensory qualities. | Canned or bottled vegetables, fruits and legumes; salted or sugared nuts and seeds; salted, cured, or smoked meats; canned fish; fruits in syrup; cheeses and unpackaged freshly made breads; alcoholic drinks produced by fermentation of group 1 foods, such as beer, cider and wine. | Additives can be used to preserve their original properties or to resist microbial contamination. For example, fruit in syrup with added antioxidants and dried salted meats with added preservatives |
|  | **Definition** | **Processes used** | **Process objectives** | **Examples** | **Adds** |
| **Group 4: Ultra-processed food and drink products** | Industrial formulations typically with five or more and usually many ingredients. Such ingredients often include those also used in processed foods, such as sugar, oils, fats, salt, anti-oxidants, stabilisers and preservatives. Ingredients only found in ultra-processed products include substances not commonly used in culinary preparations, and additives whose purpose is to imitate sensory qualities of group 1 foods or of culinary preparations of these foods, or to disguise undesirable sensory qualities of the final product. Group 1 foods are a small proportion of or are even absent from ultra-processed products. | Several industrial processes with no domestic equivalents, such as extrusion and moulding, and pre-processing for frying. | Create products that are ready to eat, to drink, or to heat, liable to replace both unprocessed or minimally processed foods that are naturally ready to consume, such as fruits or nuts, milk and water, and freshly prepared drinks, dishes, desserts and meals. Common attributes of "ultra-processed" products are hyper-palatability, sophisticated and attractive packaging, multi-media and other aggressive marketing to children and adolescents, health claims, high profitability, and branding and ownership by transnational corporations. | Carbonated drinks; sweet or savoury packaged snacks; ice cream, chocolate, candies (confectionery); mass-produced packaged breads and buns; margarines and spreads; cookies (biscuits), pastries, cakes and cake mixes; breakfast “cereal”, “cereal” and “energy” bars; “energy” drinks; milk drinks, “fruit” yoghurts and “fruit” drinks, cocoa drinks; meat and chicken extracts and “instant” sauces; infant formulas, follows-on milks and other baby products; “health” and “slimming” products such as powdered or “fortified” meal and dish substitutes; and many ready-to-heat products (including pre-prepared pies and pasta and pizza dishes); poultry and fish “nuggets” and “sticks”, sausages, burgers, hot dogs, and other reconstituted meat products, and powdered and packaged instant soups, noodles and desserts.  Products made solely of group 1 or group 3 foods also containing cosmetic or sensory intensifying additives, such as plain yogurt with artificial added artificial sweeteners, and breads with added emulsifiers.  When alcoholic drinks are identified as foods, those produced by fermentation of group 1 foods followed by distillation of the resulting alcohol such as whisky, gin, rum, vodka. | Substances only found in ultra-processed products include some directly extracted from foods such as casein, lactose, whey, gluten, and some derived from further processing of food constituents, such as hydrogenated or interesterified oils, hydrolysed proteins, soy protein isolate, maltodextrin, invert sugar, high fructose corn syrup. Classes of additive only found in ultra-processed food include dyes and other colours, colour stabilisers, flavours, flavour enhancers, non-sugar sweeteners, and processing aids such as carbonating, firming, bulking and anti-bulking, de-foaming, anti-caking and glazing agents, emulsifiers, sequestrants and humectants. |
